# Human body odor increases familiarity for faces during encoding‐retrieval task

**DOI:** 10.1002/hbm.24920

**Published:** 2020-01-06

**Authors:** Cinzia Cecchetto, Florian Ph. S. Fischmeister, Sarah Gorkiewicz, Wolfgang Schuehly, Deepika Bagga, Valentina Parma, Veronika Schöpf

**Affiliations:** ^1^ Institute of Psychology, University of Graz Graz Austria; ^2^ BioTechMed Graz Austria; ^3^ Institute of Biology, University of Graz Graz Austria; ^4^ Department of Psychology Temple University Philadelphia Pennsylvania; ^5^ Computational Imaging Research Lab (CIR), Department of Biomedical Imaging and Image‐guided Therapy Medical University of Vienna Vienna Austria

**Keywords:** body odors, chemosignals, context‐dependent memory, encoding‐retrieval face, episodic memory, fMRI, olfaction

## Abstract

Odors can increase memory performance when presented as context during both encoding and retrieval phases. Since information from different sensory modalities is integrated into a unified conceptual knowledge, we hypothesize that the social information from body odors and faces would be integrated during encoding. The integration of such social information would enhance retrieval more so than when the encoding occurs in the context of common odors. To examine this hypothesis and to further explore the underlying neural correlates of this behavior, we have conducted a functional magnetic resonance imaging study in which participants performed an encoding‐retrieval memory task for faces during the presentation of common odor, body odor or clean air. At the behavioral level, results show that participants were less biased and faster in recognizing faces when presented in concomitance with the body odor compared to the common odor. At the neural level, the encoding of faces in the body odor condition, compared to common odor and clean air conditions, showed greater activation in areas related to associative memory (dorsolateral prefrontal cortex), odor perception and multisensory integration (orbitofrontal cortex). These results suggest that face and body odor information were integrated and as a result, participants were faster in recognizing previously presented material.

## 1 INTRODUCTION

Odors are effective contextual cues in the recollection or recognition of information experienced in the recent or remote past (Larsson, Arshamian, & Kärnekull, 2017). When the same odor is presented at encoding and retrieval, such odor context facilitates recollection of the information compared to when the odor context is changed between encoding and retrieval (Ball, Shoker, & Miles, 2010; Cann & Ross, 1989; Herz, 1997; Parker & Gellatly, 1997; Parker, Ngu, & Cassaday, 2001; Wiemers, Sauvage, & Wolf, 2014). In light of the tight connection between olfactory cortices and limbic areas, odors represent special contextual information. Olfactory information is directly transduced from the olfactory bulb and the piriform cortex into the amygdala and the hippocampus, without passing through the thalamus (Lundström, Boesveldt, & Albrecht, 2011; Powell, Cowan, & Raisman, 1965). This direct route plays a central role in memory and emotional processing (Delplanque, Coppin, & Sander, 2017; Kadohisa, 2013; Larsson & Willander, 2009). Surprisingly, the functional neural networks during odor context‐dependent memory are relatively unexplored, as compared to encoding and retrieval processing of visual stimuli (Spaniol et al., 2009). In a recent study from our lab, Reichert et al. (2017) observed increased activation in the piriform cortex for successfully encoded non‐social stimuli when a congruent odor was presented compared to an incongruent odor presentation condition. This may suggest an enhanced retrieval of information when previously encoded with odor presentation (Reichert et al., 2017). The involvement of the piriform cortex was also reported in a slightly different paradigm investigating cross‐modal (olfactory‐visual) recognition memory (Gottfried, Smith, Rugg, & Dolan, 2004): in this study, objects were presented with odors during memory encoding and then neural responses were examined during the recognition of the objects alone. The authors found activations within the piriform cortex and the anterior hippocampus during successful retrieval. Interestingly, the same study (Gottfried et al., 2004) established that the neural areas involved in emotional contextual retrieval were located in subregions of the orbitofrontal cortex (OFC), an area that is known to integrate olfactory information with knowledge coming from other sensory and cognitive processes (Gottfried & Zald, 2005; Seubert, Freiherr, Frasnelli, Hummel, & Lundström, 2013).

Other studies (Cann & Ross, 1989; Hackländer & Bermeitinger, 2017; Herz & Cupchik, 1995) have investigated whether affective congruency between the olfactory context and the material to be remembered can enhance memory performance. Even though these studies did not find clear evidence for the affective congruency effect in memory (Herz & Cupchik, 1995), they still raised the question of whether specific odors can be particularly effective contextual cues for the encoding‐retrieval of stimuli. Besides affective congruency, another aspect that can put two stimuli into relation is the semantic category of the transmitted information. Previous studies have provided evidence that the human brain organizes concepts based on semantic categories (Handjaras et al., 2016; Mahon, Anzellotti, Schwarzbach, Zampini, & Caramazza, 2009) across different modalities to integrate them into unified conceptual knowledge (Calvert, Campbell, & Brammer, 2000; Gottfried & Dolan, 2003; Seo & Hummel, 2017). For example, several studies have shown that the information we use for the identification of other people, such as faces and voices, is organized and combined as person‐identity information (see Belin, 2017; Blank, Wieland, & von Kriegstein, 2014 for recent reviews). If this is especially true for faces and voices (Blank et al., 2014), growing evidence suggests that even the odor of human sweat (also called body odors or chemosignals) can be considered an important source for social information. Indeed, humans transfer socially relevant information, such as age, gender, health status, sexual availability, and personal predispositions also via body odors (McClintock et al., 2005; Parma et al., 2017). Moreover, it has been shown that distinct neural pathways are responsible for the processing of body odors (Lundström, Boyle, Zatorre, & Jones‐Gotman, 2008), in line with what was found for the processing of faces (Haxby, Hoffman, & Gobbini, 2000, 2002) and voices (Belin, Fecteau, & Bédard, 2004), respectively. Processing body odors recruits the occipital cortex that becomes active when either visual stimuli or socially relevant stimuli are cross‐modally presented (Haxby et al., 2000). Other brain areas include the angular gyrus, responsive to information related to the human body (Seghier, 2013) and, the anterior and posterior cingulate cortex, previously found involved in emotion regulation (Cato et al., 2004; Johns, Inzlicht, & Schmader, 2008) and self‐reflective processes (van der Meer, Costafreda, Aleman, & David, 2010).

Based on the strong relationship between social stimuli from different sensory modalities, we hypothesize that the social information transmitted through body odors might be integrated with social information acquired during the encoding of faces to enhance subsequent retrieval. As previous evidence (Gottfried & Dolan, 2003) has shown, the detection of an odor is faster and more accurate when the odor is processed in the context of semantically congruent visual cues, therefore, we expect that an enhancement effect of an odor context in the encoding and recognition of faces will be stronger when the presented odor is a body odor compared to a non‐social common odor.

To investigate this hypothesis, we asked participants to perform an incidental‐encoding task and a yes/no recognition task. Both tasks were performed during functional magnetic resonance imaging (fMRI), by three separate groups. The BO group performed both encoding and retrieval while exposed to the chemosensory stimulus of masked body odor; the MASK group performed both encoding and retrieval while exposed to a common odor used as masker; the I‐BO group performed either encoding or retrieval while exposed to the chemosensory stimulus of masked body odor. All groups performed both tasks also while exposed to clean air as control. We hypothesize that the masked body odor presented both at encoding and at retrieval would enhance face recognition compared to the common odor or clean air. The masked body odor presented only during encoding or retrieval will help to clarify whether the integration of the social information needs to happen at the encoding phase or whether it can increase recognition also at the retrieval phase. With respect to the neural underpinnings, we speculate that, during the presentation of the body odors, there will be increased activations in areas commonly associated with social information, including body odor processing, such as the angular gyrus, occipital cortex, and the anterior and posterior cingulate cortex (Cecchetto, Lancini, Bueti, Rumiati, & Parma, 2019; Lundström et al., 2008; Parma et al., 2017) and areas involved in the integration of information coming from different sensory modalities such as the OFC (Gottfried et al., 2004; Gottfried & Zald, 2005; Seubert, Freiherr, Frasnelli, et al., 2013). Moreover, we foresee the activation of areas associated with the more general cognitive functions of encoding and retrieval success such as medial‐temporal, prefrontal (dorsolateral and anterior PFC), and parietal regions (Spaniol et al., 2009).

## 2 MATERIALS AND METHODS

### 2.1 Body odor donors

Twenty‐seven healthy, heterosexual women donated their axillary sweat (age: M = 22.11 years; *SD* = 4.32; range = 19–42 years). The donors reported: (a) to be non‐smokers; (b) not to have health issues or to undergo drug treatment known to be related to olfactory alterations; (c) not to use hormonal contraception; (d) not to be pregnant. Donors provided written informed consent and agreed to follow behavioral, nutritional (i.e., no alcohol, smoking, food altering the natural body odor), and hygiene instructions starting 2 days before the odor collection session (following Parma et al., 2017). The medium of body odor collection used was a T‐shirt washed beforehand with an odorless detergent, and with sterilized cotton pads attached to the armpit zone. Each donor wore the T‐shirt for 12 consecutive hours during the day, right after having taken a shower using fragrance‐free body wash and having dried themselves with towels washed with the same odor‐free detergent, which was the same used to pre‐wash the T‐shirts. Aluminum foil and odorless plastic bags were provided to each donor to wrap and to store the T‐shirt before bringing it back to the lab, the day following the collection period (Lundström et al., 2008; Lundström & Jones‐Gotman, 2009). Samples were perceptually evaluated for odor contamination (e.g., alcohol, smoke, fragrance, food) and for body odor detectability by a trained experimenter. All samples were then stored in a −80°C freezer for a maximum of 7 months to prevent deterioration (Lenochova, Roberts, & Havlicek, 2008). None of the donors took part in the main experiment as a participant.

### 2.2 Participants

In this study only women were included, since it has been shown that the processing of human body odor is influenced by gender (Krajnik, Kollndorfer, Nenning, Lundström, & Schöpf, 2014; Martins et al., 2005). Additionally, women present greater preference as compared to men for social emotional stimuli (Lübke, Hoenen, & Pause, 2012; Proverbio, Zani, & Adorni, 2008). By focusing on a very homogenous sample, gender‐related effects could be avoided and the power of the results would increase. Fifty‐six women were recruited for the main experiment, with the following exclusion criteria: being MRI incompatible (especially metallic implants, pacemakers, claustrophobia); being left‐handed; being pregnant; cardiovascular, neurological or psychiatric disease, diseases of the central nervous system and diseases affecting brain metabolism; taking hormonal contraception (Kollndorfer, Ohrenberger, & Schöpf, 2016); drug and nicotine consumption (Boesveldt et al., 2011); presence of olfactory dysfunction; previous head trauma leading to unconsciousness; chronic rhinosinusitis; non‐heterosexual (Krajnik et al., 2014; Martins et al., 2005).

Since previous studies have shown that olfactory perception is affected by menstrual cycle phase (Nováková, Havlíček, & Roberts, 2014; Pause, Sojka, Krauel, Fehm‐Wolfsdorf, & Ferstl, 1996), in particular for evaluations of body odors (Lundström, Olsson, Schaal, & Hummel, 2006), each participant cycle's length was standardized to a 28‐day cycle (see Data S1) and compared across groups (see Section 3). All participants were instructed to avoid eating and drinking anything other than water 1 hr prior to testing and using any scented products on the day of the study. The study was approved by the local ethics committee of the University of Graz (Austria) in accordance with the 1975 Declaration of Helsinki and its later amendments. All participants provided written informed consent before participation in the study. Participants were compensated with course credits or money compensation in line with the university standards.

### 2.3 Pre‐screening

In order to be included in the main study, participants were pre‐screened for normative olfactory function and normal face perception. Olfactory function was assessed with the computer‐testing version of the standardized clinically approved “Sniffin' Sticks” test (Burghart Instruments, Wedel, Germany; Hummel et al., 1997). Three different olfactory functions were assessed: First, the odor detection threshold was determined for *n*‐butanol with 16 stepwise dilutions using an ascending limits procedure (Sijben, Panzram, Rodriguez‐Raecke, Haarmeier, & Freiherr, 2018) based on a three‐alternative forced choice task (3AFC). Second, odor discrimination was assessed over 16 trials again using a 3AFC task. For each discrimination step, three pens were presented in random order; two containing the same odor and the third containing the target odor. Third, odor identification was measured by presenting 16 common odors, each presented with four verbal descriptors in a multiple forced‐choice format (three distractors and one target). A total score TDI above 30.5 was considered to be within the normosmic range (Hummel et al., 2007). Only participants obtaining a TDI score in the normosmic range were included in the study.

Face perception abilities were assessed with the upright and inverted versions of the Cambridge Face Memory Test (CFMT, Daini, Comparetti, & Ricciardelli, 2014; Duchaine & Nakayama, 2006). This test is composed of 72 trials divided in three stages of increasing difficulty. Participants memorize six unfamiliar target faces, and are then required to recognize them from sets of three faces (one target and two distractor faces). Only participants with a CFMT score above the cut‐off (52 score) were included in the study.

### 2.4 Stimuli

Participants were randomly distributed to a congruent BO group exposed to masked body odor, a congruent MASK group exposed to the masker odor only, and an incongruent I‐BO group exposed to the masked body odor only during the encoding or the recognition task. The masker odor was an emotionally neutral, rather unfamiliar odor (1% vigoflor, International Flavors & Fragrances, New York, CAS 68480‐11‐5, in 1,2‐propylene glycol) placed in a jar with four clean cotton pad quadrants. This concentration was determined via pilot studies (*N* = 19 participants) as less familiar, more pleasant and less arousing than the other odors (see Data S1). The vigoflor solution was also used as masker odor for the other two odor conditions (BO and I‐BO) to perceptually mask the body odor samples. Masking was done to increase consistency across samples and to avoid that individual odors would be recognized and exert specific influence (Cecchetto, Lancini, Bueti, et al., 2019; Martins et al., 2005; Wudarczyk et al., 2015). Masked body odor was prepared by placing in a jar four cotton pads quadrants from four donated T‐shirts chosen from all those collected from the 27 donors and one clean cotton pad quadrant on which we applied 0.2 ml of masker odor (Martins et al., 2005). In each jar, two quadrants were from the left axilla and two from the right (Mitro, Gordon, Olsson, Lundström, & Vainius, 2012). The masking procedure was used to simulate the hygiene products usually used with the goal of making the paradigm more ecologically valid.

A total of 246 neutral faces was selected from the Chicago face database (Ma, Correll, & Wittenbrink, 2015) for the encoding‐retrieval memory task. These faces were distributed in four groups of 60 stimuli each; two groups were used during encoding and two during retrieval. The images were distributed in order to match for physical facial features, for age of the actors, attractiveness, femininity, masculinity, trustworthiness and for the seven level of emotional expressiveness (ratings were provided in Ma et al., 2015). The remaining six faces were used for training purposes. All faces were cropped to remove hair, neck and shoulders, and then presented on a white background. The pictures were finally converted to a size of 1152 (width) by 864 (height) pixels.

### 2.5 Odor ratings and mood assessment

Before and after the picture encoding‐retrieval memory tasks, participants completed an odor rating task and filled questionnaires regarding their affective state (as assessed with the Positive and Negative Affect Schedule, PANAS; Watson, Clark, & Tellegen, 1988; German version, Janke & Glöckner‐Rist, 2014 and anxiety state Spielberger, 1968). Information regarding affective and anxiety states were collected because previous studies have shown that olfactory perception can be modulated by emotional states (Kadohisa, 2013; Krusemark, Novak, Gitelman, & Li, 2013; Takahashi et al., 2015; see Data S1 for the results of mood assessment). The odor rating was done to familiarize all participants with the masked body odor or with the masker odor that was used in the subsequent stages of the study (compare Reichert et al., 2017) and to make sure that the masking procedure worked. In order to avoid that participants focused their attention on the odor used during encoding‐retrieval task, they were asked to rate intensity, pleasantness, arousal, and familiarity of four odors (the masked body odor or the masker odor and in addition 1% cedarwood oil in 1,2‐propylene glycol, 1% eugenol in 1,2‐propylene glycol and clean air). Odor ratings were collected on a 9‐point visual analog scale, ranging from “not at all” to “very much.” Participants were instructed to close their eyes during the odor presentation and to answer even when they did not perceive an odor.

### 2.6 Encoding‐retrieval task

**Figure 1:**
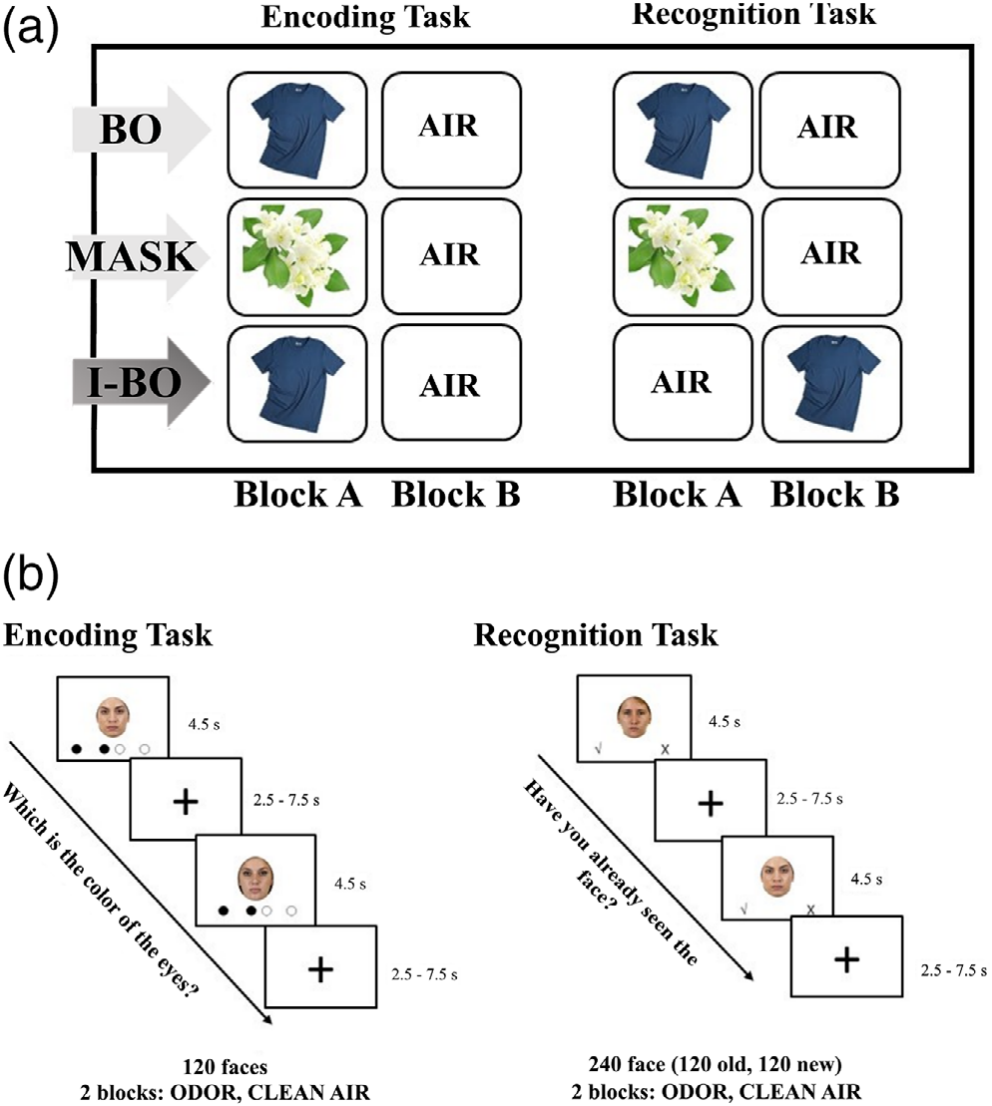
(a) Overview of the study procedures with odor presentation per block and study group. BO, congruent group exposed to masked body odor; I‐BO, incongruent group exposed to the masked body odor; MASK, congruent group exposed to the masker odor. (b) Stimulus timing for encoding and recognition task. Inter‐stimulus intervals were jittered as shown (duration 2.5–7.5 s). During the encoding task, participants evaluated whether the color of the eyes of each face was dark (index finger), light (ring finger) or a mixed color (middle finger button press). During the recognition task, participants evaluated by button press whether or not they had previously seen the face in the encoding task (yes = index finger, no = ring finger)

After the initial odor rating and mood assessment, participants performed the face encoding‐retrieval task. This task consisted of two fMRI sessions: an incidental‐encoding task, followed by simple yes/no recognition task, with a retention interval in between. Each task was composed of two blocks (A and B) each one characterized by a different odor condition (see Figure 1a for details). Before each task, participants were prepared and instructed. As a cover story for the task, participants were informed that the study investigated the influence of odors on face perception.

In the incidental‐encoding task, 60 faces were presented in each block. In Block A, faces were presented simultaneously with the masked body odor presentation for the BO and I‐BO groups or with the masker odor for the MASK group. In Block B, faces were presented with clean air administered to all three groups. The order of blocks as well as the order of the trials within each block were randomized per participant. Participants evaluated by button press whether the color of the eyes of each face was dark, light or a mixed color (see Figure 1b). Just before the encoding and recognition tasks, six training trials were performed to practice the use of the response buttons.

The incidental‐encoding task was followed by a retention time interval, spent outside of the scanner in a quiet room, of approximately 50 min (mean = 55 min, *SD* = 8 min). During this time, questionnaires regarding the individual significance of the sense of smell (IOQ, Croy, Buschhüter, Seo, Negoias, & Hummel, 2010), current symptom levels of depression (BDI, German version, Beck, Steer, & Hautzinger, 1995) and alexithymia (BVAQ, Cecchetto, Rumiati, & Aiello, 2017; Vorst & Bermond, 2001) were conducted.

Afterwards, the yes/no recognition task was conducted. Each block of the recognition task included 120 stimuli (the 60 previously shown faces from the encoding task and 60 new faces), for a total of 240 faces. During Block A, faces were presented in concomitant with the masked body odor to the BO group, the masker odor was presented to the MASK group, and odorless air to I‐BO group. In Block B, faces were presented with odorless air to BO and MASK groups, while the masked body odor was presented to the I‐BO group. Again, the order of blocks and the order of trials included in each block were randomized for each participant. Participants had to indicate by button press whether they had previously seen each face (see Figure 1b). The computer‐based tasks were operated by the software Presentation (Version 18.0, Neurobehavioral Systems, Inc., Berkeley, California, http://www.neurobs.com).

During the encoding and the recognition tasks, odors were presented using an MRI compatible olfactometer described in detail by Lundström, Gordon, Alden, Boesveldt, and Albrecht (2010). In brief, the airflow through the olfactometer is controlled by solenoid valves, which direct the odors through the odor glass reservoir (for odor block) or an empty glass reservoir (for clean air block). The air used to operate the olfactometer was filtered using active carbon to avoid contamination by residual odors. A continuous odorless airstream of 1 L/min was transported to the birhinal nosepiece, masking tactile cues that might otherwise result from channel opening. Odorous or odorless air (3.5 L/min) was directed to the nose when the faces were presented. After odor presentation, clean air was presented until participant's response to minimize odor residuals (Seubert, Gregory, Chamberland, Dessirier, & Lundström, 2014). Odor stimuli were delivered directly to both nostrils from a nasal manifold, attached to the participant's chest by means of a chest strap, connected to the olfactometer via teflon tubing.

### 2.7 MRI image acquisition

Functional and anatomical images were acquired with a 3‐Tesla MRI scanner (Skyra, Siemens, Erlangen, Germany) using a 32‐channel head coil. Eighty axial slices were acquired using a multiband EPI sequence (TR = 1.40 s, TE = 30 ms, FA = 65°, FoV = 220 × 220 mm^2^, matrix size = 110 × 110, voxel size =2 × 2 × 2 mm^3^, multi‐band factor: 4, in‐plane acceleration [GRAPPA] = 2). The number of volumes acquired varied for each participant and run based on the task duration based on participants' reaction times (Encoding: Run A = 427.94 ± 15.10; Run B = 423.44 ± 17.43; Recognition: Run A = 841.41 ± 47.62; Run B = 836.22 ± 36.44). T1‐weighted 3D gradient echo sequence scans (MPRAGE, 176 sagittal slices, TR = 2.53 s, TE = 2.26 ms, TI = 9 ms, slice thickness = 1 mm, FoV = 256 × 256 mm^2^) were acquired coplanar with the functional scans. For field‐mapping a multi‐echo GE images were acquired once before the functional runs with geometry and orientation matching that of the functional runs. The remaining sequence parameters were: TE = (4.92, 7.38) ms, TR = 791 ms, FA = 60°, RBW = 590 Hz/pixel.

### 2.8 Behavioral data analysis

Behavioral analyses were performed with Linear Mixed Models (LMMs) with intercepts for participants as random effect to account for the high variability across individuals. This type of analysis reduces Type I errors and allows for the generalization of findings to other samples of participants (Judd, Westfall, & Kenny, 2012). LMMs were fitted and analyzed using R (version 2.10.1; http://www.r-project.org/) and in particular using the *lme* function from the *nlme* package (https://cran.r-project.org/web/packages/nlme/nlme.pdf). For post‐hoc comparisons of significant interactions, the *lsmeans* package was used. As a measure of goodness of fit of the chosen LMMs, we also report conditional *R* ^2^, which describes the proportion of variance explained by both the fixed and random factors (Johnson, 2014).

To compare socio‐demographic and questionnaires scores across groups, LMM were built with group as the only fixed effect factor. All the variables that were significantly affected by the group factor were included in the following LMMs as additional fixed factor and in the second level neuroimaging analysis as covariate. For the analysis of odor rating, LMMs were separately built for each dependent variable (intensity, arousal, familiarity and pleasantness). The LMMs included a three‐way interaction with groups, time points (pre‐ and post‐task) and odor condition (masked body odor/masker odor and clean air were the only odors considered for the analysis as were the ones used during the tasks). For the picture recognition task, hit rates (the probability of correctly recognizing old faces), false alarm rates (the probability of incorrectly recognizing new faces as old), *d*′ (the ability to discriminate between new and old faces), response bias (the general tendency to respond with yes or no) were calculated according to signal detection theory (Stanislaw & Todorov, 1999) for Block A and Block B and for the three groups separately. LMMs were performed for each behavioral task performance (hit rates, false alarm rates, *d*′, response bias and reaction times) with interaction of groups and blocks as fixed factor. Moreover, since our main hypothesis focused on the contrast between the two congruent groups, MASK and BO, and on the effect of the body odor on the incidental‐encoding task, to better explore this contrast, independent *t* tests were performed between the two groups in the odor block and in the air block. Finally, to explore whether the task has different effects on the mood of the three groups, LMMs were performed on STAI and PANAS positive and negative scores with the interaction of groups and time points (pre‐ and post‐task) as fixed factor.

### 2.9 MRI data preprocessing

Results included in this manuscript come from preprocessing performed using fMRIPrep 1.1.3 (Esteban et al., 2019), which is based on Nipype 1.1.1 (Gorgolewski et al., 2011, 2018). fMRIPrep represents an analysis‐agnostic preprocessing pipeline allowing for a robust, convenient and reproducible preprocessing of data without the need for manual interference. This toolbox combines already available methods implemented in software‐packages available to the public to form an optimized transparent workflow.

The T1‐weighted (T1w) image was corrected for intensity non‐uniformity using *N4BiasFieldCorrection* (Tustison et al., 2010; ANTs 2.2.0), and used as T1w‐reference throughout the workflow. The T1w‐reference was then skull‐stripped using *antsBrainExtraction.sh* (ANTs 2.2.0), using OASIS as target template. Spatial normalization to the ICBM 152 Nonlinear Asymmetrical template version 2009c (Fonov, Evans, McKinstry, Almli, & Collins, 2009) was performed through nonlinear registration with *antsRegistration* (ANTs 2.2.0; Avants, Epstein, Grossman, & Geea, 2008), using brain‐extracted versions of both T1w volume and template. Brain tissue segmentation of cerebrospinal fluid, white matter (WM) and gray matter (GM) was performed on the brain‐extracted T1w using *fast* (FSL 5.0.9; Zhang, Brady, & Smith, 2001).

For each of the four functional BOLD runs found per subject (Blocks A and B within the encoding and the recognition task), the following preprocessing was performed. First, a reference volume and its skull‐stripped version were generated using a custom methodology of fMRIPrep. A deformation field to correct for susceptibility distortions was estimated based on a field map that was co‐registered to the BOLD reference, using a custom workflow of fMRIPrep derived from D. Greve's *epidewarp.fsl* script and further improvements of HCP Pipelines (Glasser et al., 2013). Based on the estimated susceptibility distortion, an unwrapped BOLD reference was calculated for a more accurate co‐registration with the anatomical reference. Head‐motion parameters with respect to the BOLD reference (transformation matrices, together with the six corresponding rotation and translation parameters) were estimated before any spatiotemporal filtering using *mcflirt* (FSL 5.0.9; Jenkinson, Bannister, Brady, & Smith, 2002). BOLD runs were slice‐time corrected using 3dTshift from AFNI (Cox & Hyde, 1997). The BOLD time‐series were resampled onto their original, native space by applying a single, composite transform to correct for head‐motion and susceptibility distortions. These resampled BOLD time‐series will be referred to as *preprocessed BOLD in original space*, or just *preprocessed BOLD*. The BOLD reference was then co‐registered to the T1w reference using *flirt* (FSL 5.0.9; Jenkinson & Smith, 2001) with the boundary‐based registration (Greve & Fischl, 2009) cost‐function. Co‐registration was configured with nine degrees of freedom to account for distortions remaining in the BOLD reference. The BOLD time‐series were resampled to MNI152NLin2009cAsym standard space, generating a preprocessed BOLD run in MNI152NLin2009cAsym space. All functional volumes were then smoothed using a Gaussian kernel with full width at half maximum of 8 mm^3^ using SPM12 (Wellcome Trust Centre for Neuroimaging, London, UK).

### 2.10 MRI data analysis

Statistical analyses were based on a previous study of our group by Reichert et al. (2017) and were performed using a general linear model approach as implemented in SPM12. In the first‐level analysis, data were analyzed separately for each participant. For the encoding task, the classification of trials was based on the subsequent performance at the recognition task. Therefore, the conditions subsequent hits (trials in which the face was correctly recognized as old in the recognition task) and subsequent misses (trials in which the face was not recognized as old in the recognition task) were modeled as regressors of interest. For the recognition task, the conditions hits (trials in which the old face was correctly recognized as old), false alarms (trials in which the new face was recognized as old), correct rejections (trials in which the new face was correctly recognized as new), and misses (trials in which the old face was not recognized as old) were modeled. For both tasks, button press onset and realignment parameters consisting of three rotations and three translations in space were included as regressors of no interest. All task regressors were convolved with a canonical hemodynamic response function. Low frequency signal drifts were filtered using a cutoff period of 128 s. As a next step, at the individual level, single contrasts were performed for subsequent hits and subsequent misses again for both odor and air blocks for the encoding task; for the recognition task, single contrasts were done for hits and false alarms for both odor and air blocks.

Subsequently, at the second‐level analysis, the resulting contrast images were submitted to four separated full factorial models (hits and misses for encoding task and hits and false alarms for recognition task). These full factorial designs consisted of one within‐subject factor (odor condition) and one between subject factor (group) to investigate main effects and interactions separately. In each analysis, BDI scores (see Section 3.1) were inserted as covariates. To identify the neuronal substrates of single odor condition, simple main effects (i.e., [odor − air] for each group) were analyzed. To investigate whether odor conditions affect neural activity related to group, we performed two odor conditions (odor/air) by group (BO/MASK or BO/I‐BO) interactions. To investigate the effect of congruency independently of the odor condition, we analyzed the contrast (BO − I‐BO) averaged over odor and air condition. Finally, to investigate the odor effect independently of the type of odor, we analyzed the contrast [odor −air] across all groups.

Whole‐brain statistical maps were corrected with a *p* = .001 cluster‐forming threshold and then a cluster‐threshold of *p* = .05 family‐wise error correction was applied. The coordinates of resulting activations are presented in MNI space. The anatomical location of peaks was determined on the basis of the neuromorphometrics atlas in SPM (Neuromorphometrics, Inc.; http://neuromorphometrics.com/).

## 3 RESULTS

### 3.1 Demographic information and questionnaires

From the initial sample, two participants were removed. One person responded only to 58 trials (on 120 total) in the recognition task and she gave all “no” button responses; the other showed signs of severe depression according to the BDI questionnaire (Beck, Steer, & Brown, 1996). The final size was 54 participants (see Table 1 for means and *SD* of demographic variables and questionnaire scores). LMM with group as factor showed no significant results when applied on age (all *β* < 1.78, all *SE* > 1.30, all *t* < 1.37, all *p* > .17), standardized menstrual cycle (all *β* < 116.81, all *SE* > 8.73, all *t* < 19.17, all *p* > .05), score at CFMT (all *β* < 59.76, all *SE* > 1.71, all *t* < 49.83, all *p* > .36), total score of IOQ (all *β* < 0.02, all *SE* > 0.18, all *t* < 0.10, all *p* > .25), alexithymia (BVAQ, Vorst & Bermond, 2001; all *β* < 1.99, all *SE* > 3.63, all *t* < 0.38, all *p* > .25), and TDI score (all *β* < 0.64, all *SE* > 0.90, all *t* < 0.69, all *p* > .48). The LMM with group as factor on BDI score (AIC = 307.39; BIC = 317.05; logLik = −148.69; *R* ^2^ = .89) showed that the BDI score was significantly different between groups with the MASK group presenting higher BDI score than BO group (*β* = 3.41, *SE* = 1.37, *t* = 2.49, *p* = .016) but no significant differences between the groups BO and I‐BO (*β* = 2.44, *SE* = 1.35, *t* = 1.81, *p* = .08) and between MASK and I‐BO (*β* = −0.96, *SE* = 1.39, *t* = −0.69, *p* = .49). Therefore, BDI was inserted as fixed factor in the following behavioral analysis and as covariate in the neuroimaging analysis.

**Table 1:** Demographic characteristics and questionnaire scores of the three groups

Group	*n*	Age (years)	Standard menstrual cycle	TDI	CFMT	IOQ	BDI	BVAQ
BO	19	24.21 (2.84)	13.68 (8.66)	34.47 (2.17)	83.00 (7.04)	117.63 (14.07)	3.00 (3.49)	88.84 (18.42)
MASK	17	26.00 (5.61)	15.05 (8.02)	35.12 (7.21)	80.76 (7.21)	118.35 (7.39)	6.41 (4.19)	82.70 (14.41)
I‐BO	18	22.61 (2.77)	23.71 (27.78)	34.11 (3.04)	81.11 (7.53)	110 (30.33)	5.44 (4.57)	90.83 (14.07)
Range	—	18–39	0–28	0–48	0–72	40–160	0–63	40–200

*Note*: Mean values and *SD*s (in brackets) are given for age, standardized menstrual cycle, importance of olfaction (IOQ, Croy et al., 2010), depression scale (BDI, Beck et al., 1995) and alexithymia (BVAQ, Vorst & Bermond, 2001). BO, congruent group exposed to masked body odor; I‐BO, incongruent group exposed to the masked body odor; MASK, congruent group exposed to the masker odor.

### 3.2 Odor rating task

Odor rating data allowed us to test whether the masking procedure applied to cover the body odor rendered the odor conditions (masked body odor and masker odor) equivalent in their basic perceptual dimensions. The LMM on intensity rating (AIC = 841.758; BIC = 875.51; logLik = −410.88; *R* ^2^ = 0.65) revealed a significant effect of odor condition: clean air was perceived as less intense than masker and masked body odor (*β* = 3.26, *SE* = 0.34, *t* = 9.34, *p* < .001). Moreover, the odor condition significantly interacted with the MASK group (*β* = 1.03, *SE* = 0.51, *t* = 2.03, *p* = .04), however, the post‐hoc test (*lsmeans* function) showed no significant contrasts between masker and masked body odor (all *p* > .11). No effect of time points or group were retrieved (all *β* < 3.26, all *SE* > 0.03, all *t* < 9.34, all *p* > .17).

The LMM on familiarity rating (AIC = 969.32; BIC = 1,009.82; logLik = −472.66; *R* ^2^ = .26) showed a significant main effect of odor condition (*β* = 2.59, *SE* = 0.52, *t* = 4.95, *p* < .001): clean air was perceived as less familiar than masker and masked body odor. Moreover, there was a significant main effect of time (*β* = 1.47, *SE* = 0.52, *t* = 2.81, *p* = .005): as expected, both odors and clean air were perceived as more familiar in the post‐task session than pre‐task. Finally, there was a significant main effect of group: MASK group rated as less familiar both odor and clean air than BO group (*β* = 1.35, *SE* = 0.66, *t* = 2.05, *p* = .046) and I‐BO group (*β* = 1.38, *SE* = 0.65, *t* = 2.10, *p* = .040). However, since no significant interactions between group and odor condition or between group and time were found, the group effect is not be related to the masking procedure.

The LMM on arousal rating (AIC = 861.97; BIC = 902.47; logLik = −418.98; *R* ^2^ = .41) showed a significant main effect of odor condition (*β* = 2.88, *SE* = 0.39, *t* = 7.29, *p* < .001): clean air was perceived as less arousing than mask and masked body odor. No other main effects or significant interactions were found.

The LMM on pleasantness rating (AIC = 893.45; BIC = 927.20; logLik = −436.72; *R* ^2^ = 0.24) showed no main effects or significant interactions (all *β* < 0.54, all *SE* > 0.03, all *t* < 1.07, all *p* > .08). See Table S1 for means and *SD*s of odor ratings.

### 3.3 Behavioral results: Comparison between the three groups and blocks

No significant results were found from the LMMs on hit rate (all *β* < 0.03, all *SE* > 0.004, all *t* < 0.75, all *p* > .31), false alarm rate (all *β* < 0.02, all *SE* > 0.004, all *t* < 0.64, all *p* > .28) and bias (all *β* < 0.18, all *SE* > 0.008, all *t* < 0.64, all *p* > .28). The LMM on *d*′ (AIC = 46.24; BIC = 70.38; logLik = −14.12; *R* ^2^ = .56) showed significant interactions between MASK and BO groups and blocks (*β* = 0.21, *SE* = 0.09, *t* = 2.10, *p* = .04) and between MASK and I‐BO and Blocks (*β* = 0.27, *SE* = 0.10, *t* = 2.65, *p* = .01) however the post‐hoc analysis revealed no significant contrasts. The LMM on the reaction times of the hits (AIC = −119.61; BIC = −95.47; logLik = 68.80; *R* ^2^ = .79) revealed a main effect of group (*β* = 0.12, *SE* = 0.06, *t* = 2.07, *p* = .04): BO group was faster than MASK group. The LMM on the reaction times of the false alarms (AIC = −77.62; BIC = −53.48; logLik = 47.81; *R* ^2^ = .75) revealed a main effect of group (*β* = 0.16, *SE* = 0.06, *t* = 2.40, *p* = .02): BO group was faster than MASK group. Moreover, there was a significant interaction between groups MASK and BO and blocks (*β* = −0.09, *SE* = 0.05, *t* = −2.07, *p* = .04): the post‐hoc revealed that the BO group was significantly faster than the MASK group during the odor block (*p* = .02) but not during the air block and the MASK group was significantly faster during the odor block than the air block (*p* = .047). See Table 2 for mean values and *SD*s (in brackets) of behavioral performance.

**Table 2:** Mean values and *SD*s (in brackets) of behavioral performance of picture recognition task

Groups	Hit rate	FA rate	*d*′	Bias	Hit rate RT	FA rate RT
Odor block						
BO	0.45 (0.16)	0.37 (0.12)	0.21 (0.29)	1.03 (0.11)	1,334.3 (147.4)	1,364.2 (184.6)
MASK	0.45 (0.14)	0.32 (0.16)	0.38 (0.30)	1.21 (0.34)	1,460.6 (162.6)	1,532.9 (184.5)
I‐BO	0.45 (0.18)	0.37 (0.18)	0.24 (0.26)	1.13 (0.29)	1,387.2 (176.3)	1,410.5 (200.4)
Air block						
BO	0.47 (0.15)	0.36 (0.13)	0.28 (0.30)	1.04 (0.09)	1,342.8 (186.7)	1,392.0 (205.6)
MASK	0.43 (0.11)	0.34 (0.11)	0.24 (0.32)	1.10 (0.19)	1,462.5 (179.5)	1,462.8 (223.8)
I‐BO	0.50 (0.14)	0.37 (0.17)	0.37 (0.37)	1.20 (0.51)	1,403.4 (129.7)	1,416.7 (158.6)

*Note*: BO, congruent group exposed to masked body odor; I‐BO, incongruent group exposed to the masked body odor; MASK, congruent group exposed to the masker odor.

### 3.4 Behavioral results: Comparison between congruent groups

**Figure 2:**
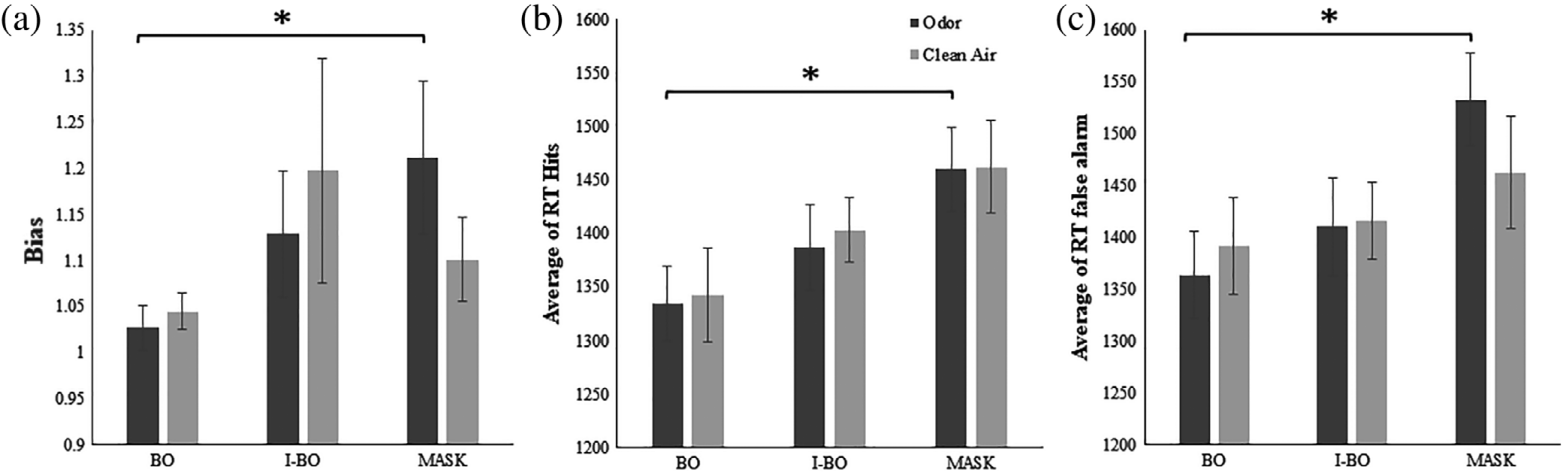
Mean values per congruent groups and odor and air blocks of (a) bias, (b) reaction time of the hits, and (c) reaction times of the false alarms. Error bars represent *SEM*

To better explore differences between the congruent groups within the odor block and the air block, two *t* tests for each behavioral task performance (hit rates, false alarm rates, *d*′, response bias and RTs) were performed between the two groups. No significant differences were found for hit rates (odor block: *t* = −0.06, *p* = .95; air block: *t* = 0.93, *p* = .35), false alarm rates (odor block: *t* = 1.04, *p* = .31; air block: *t* = 0.59, *p* = .55) and *d*′ (odor block: *t* = −1.74, *p* = .08; air block: *t* = 0.34, *p* = .73). However, the two groups were significantly different in their response bias in the odor block (*t* = −2.14, *p* = .04) but not in the air block (*t* = −1.13, *p* = .27) as well as for reaction times for hits in the odor block (*t* = −2.43, *p* = .02) but not in the air block (*t* = −1.96, *p* = .06) and for reaction times of false alarms in the odor block (*t* = −2.73, *p* = .009) but not in the air block (*t* = −0.98, *p* = .33; see Figure 2).

### 3.5 Imaging results: Encoding task

**Figure 3:**
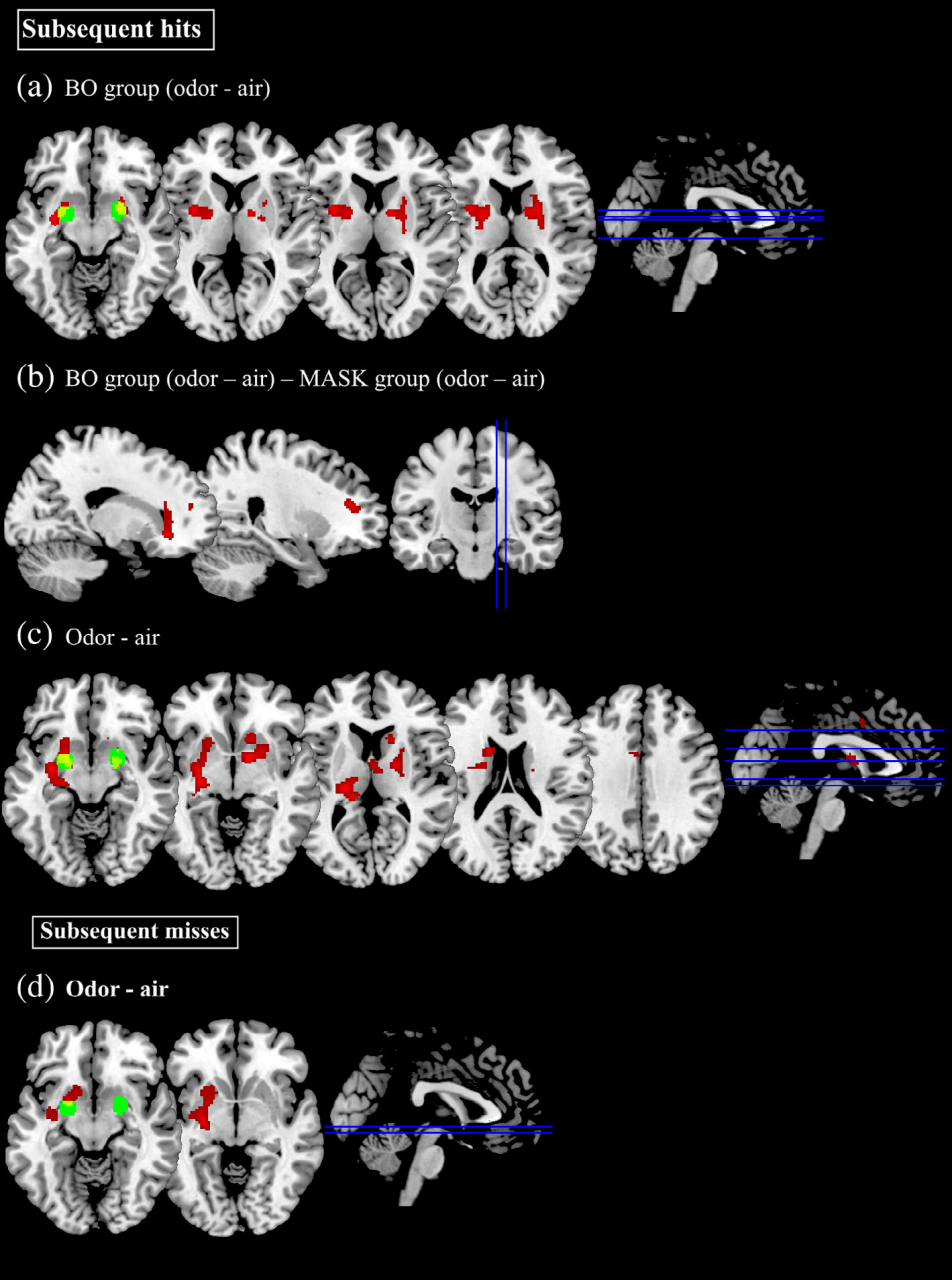
Brain activation maps showing significant cluster of activation for the encoding phase. Statistical maps are derived with a threshold of *p* < .05 FWE corrected and superimposed on a standard T1 template. Red areas = significant cluster of activation; green areas = piriform cortex activation previously reported in the literature on a meta‐analysis of olfactory studies (Seubert, Freiherr, Frasnelli, et al., 2013); yellow areas = overlap between cluster of activation and piriform cortex activation. BO, congruent group exposed to masked body odor; MASK, congruent group exposed to the masker odor

As described before, we compared subsequent hits and subsequent misses (with respect to the retrieval task) between odor and air blocks for each group and across groups (see Table 3 and Figure 3). The analysis of subsequent hits revealed the following significant clusters: the interaction [BO group (odor − air) × MASK group (odor − air)] revealed activation in the OFC and dorsolateral prefrontal cortex (dlPFC), but we did not observe any activation in these two areas for the main effect comparing (odor – air) within the BO group and the I‐BO group. However, the comparison (odor – air) within the BO group showed increased activation in the left putamen, bilateral caudate nucleus, right thalamus, and right piriform cortex; the comparison (odor – air) within the I‐BO group revealed increased activations in left superior frontal gyrus, middle frontal gyrus, putamen, hippocampus and piriform cortex. Finally, the comparison (odor – air) across all groups showed significant activations in the right pallidum, bilateral putamen, left thalamus and bilateral piriform cortex. Since the piriform cortex is not included in most standard brain atlases, the activations were compared to piriform cortex activity as reported in the literature (see Seubert, Freiherr, Frasnelli, et al., 2013).

**Table 3:** Significant clusters of neuronal activation during the encoding task

Brain region	Side	Cluster size	Peak MNI coordinates			*p* (FWE corrected)	*T*	*Z* score
			*x*	*y*	*z*			
Subsequent hits								
BO group (odor − air) × MASK group (odor − air)								
OFC	R	276	18	30	−6	.028	4.97	4.69
Middle frontal gyrus (dlPFC)	R		24	38	18		4.42	4.21
Middle frontal gyrus	R		20	46	16		4.01	3.85
BO group (odor − air)								
Putamen	L	726	−30	−4	−10	<.0001	4.68	4.45
Caudate	L		−18	6	20		4.27	4.09
Caudate	R	399	20	4	14	.006	4.52	4.31
Thalamus	R		14	0	8		4.35	4.16
Piriform cortex	R		24	4	14		3.95	3.80
I‐BO (odor − air)								
Superior frontal gyrus	L	850	−20	56	8	<.0001	5.10	4.80
Middle frontal gyrus	L		−30	62	4		4.56	4.33
Putamen	L	354	−26	−12	−4	.010	4.99	4.71
Hippocampus	L		−18	−18	−12		4.21	4.03
Piriform cortex	L		−16	−4	−12		1.06	3.90
Odor – air								
Pallidum	R	677	12	0	−2	<.0001	5.37	5.02
Putamen	R		20	−4	10		4.82	4.56
Piriform cortex	R		22	6	−4		4.56	4.34
Putamen	L	1,665	−30	−8	−8	<.0001	5.35	5.01
Thalamus	L		−20	−22	12		5.30	4.96
Piriform cortex	L		−26	−18	−6		5.25	4.92
Subsequent misses								
Odor – air								
Pallidum	L	719	−26	−10	−2	<.0001	5.43	5.08
Putamen	L		−24	4	0		4.49	4.28

*Note*: BO, congruent group exposed to masked body odor; dlPFC, dorsolateral prefrontal cortex; I‐BO, incongruent group exposed to the masked body odor; MASK, congruent group exposed to the masker odor; OFC, orbitofrontal cortex. Maximum peaks of each cluster are in first line, other maxima within the same cluster are reported in the indented lines. Peak locations are expressed in MNI coordinates. Voxelwise threshold, *p* < .001. FWE cluster level corrected *p* < .05.

The analysis of subsequent misses revealed significant increased activation for the comparison (odor – air) across all groups in the left pallidum and left putamen.

### 3.6 Imaging results: Recognition task

**Figure 4:**
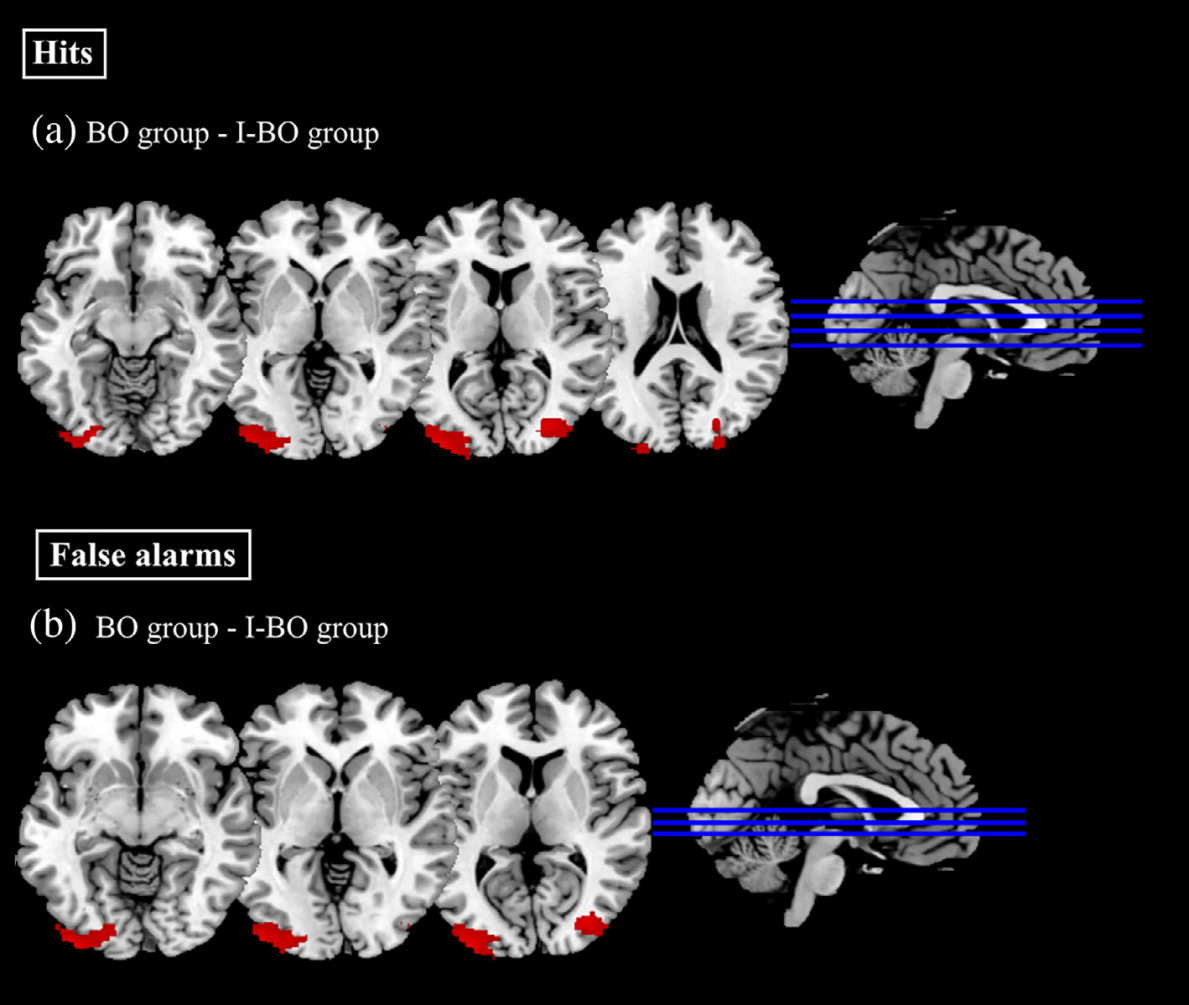
Brain activation maps showing significant cluster of activation for the recognition phase. Statistical maps are derived with a threshold of *p* < .05 FWE corrected and superimposed on a standard T1 template. BO, congruent group exposed to masked body odor; I‐BO, incongruent group exposed to the masked body odor

As described before, we compared hits and false alarms between odor and air blocks for each group separately as well as across groups (see Table 4 and Figure 4). The analysis of hits showed significant clusters for the comparison (BO group – I‐BO group) in the occipital fusiform gyrus, inferior occipital gyrus, middle and superior occipital gyrus. Finally, the analysis of false alarms for the comparison (BO group – I‐BO group) revealed significant activation in occipital fusiform gyrus, middle and inferior occipital gyrus, and superior occipital gyrus. For the recognition task, the comparisons (odor –air) within the BO group, within the I‐BO group, as well as the interaction [BO group (odor – air) × MASK group (odor – air)] for both hits and false alarms revealed nonsignificant results.

**Table 4:** Significant clusters of neuronal activation during the recognition task

Brain region	Side	Cluster size	Peak MNI coordinates			*p* (FWE corrected)	*T*	*Z* score
			*x*	*y*	*z*			
Hits								
BO group − I‐BO group								
Occipital fusiform gyrus	L	1,408	−26	−80	−16	<.0001	4.65	4.41
Inferior occipital gyrus	L		−38	−80	−18		4.50	4.28
Middle occipital gyrus	R	436	30	−82	10	.001	4.13	3.96
Superior occipital gyrus	R		26	−96	22		3.71	3.58
False alarms								
BO group − I‐BO group								
Occipital fusiform gyrus	L	1,323	−26	−80	−16	<.0001	4.62	4.39
Inferior fusiform gyrus	L		−38	−80	−18		4.49	4.27
Middle occipital gyrus	L		−38	−96	−6		4.13	3.96
Middle occipital gyrus	R	421	30	−82	10	.001	4.14	3.97
Superior occipital gyrus	R		26	−96	22		3.71	3.59

*Note*: BO, congruent group exposed to masked body odor; I‐BO, incongruent group exposed to the masked body odor; MASK, congruent group exposed to the masker odor. Maximum peaks of each cluster are in first line, other maxima within the same cluster are reported in the indented lines. Peak locations are expressed in MNI coordinates. Voxelwise threshold, *p* < .001. FWE cluster level corrected *p* < .05.

## 4 DISCUSSION

The current study aimed at investigating whether a masked human body odor, which has been shown to be able to convey social information, presented as contextual cue during encoding and recognition tasks, increased memory performance for faces in a more effective way than a neutral common odor. The results revealed that the exposure to human body odors (a) exerts behavioral effects, although they were not specific to the hit answers and (b) it was linked to stronger activity in OFC and dlPFC during the encoding phase of face pictures, suggesting the integration of the social information transmitted through images and odors.

### 4.1 Human body odors showed a facilitation effect on face recognition

As predicted, human body odors presented as contextual cue promoted enhanced memory for faces than common odors. Even though no significant differences were found for the recognition accuracy, the analysis revealed that the BO group during the odor condition, exposed to the human body odor was less biased toward the “no” response (mean *β* values closer to 1; Stanislaw & Todorov, 1999) and gave faster “yes” answers (hits rates and false alarms) compared to the MASK group. This difference was not found between the same groups in the clean air condition. This result suggests that human body odor presented as contextual cue may induce participants to recognize face images. In this sense, in the encoding task the human body odor might activate associative information that is integrated with the visual information of the face into a whole unit. During the recognition task, when the two pieces of information are repeated together, the whole unit appears more familiar (Yonelinas, 2001). However, our results showed that the facilitation effect was not specific to the hit answers (an old face is correctly recognized as “old”), but it applies also to the false alarms (a new face is identified as “old”). Since shorter reaction times indicate greater certainty in the decision process (Petrusic & Baranski, 2003), we might speculate that the human body odors, as context, represent an additional help to increment the recognition confidence for those stimuli that seemed merely familiar. Additionally, participants might be induced to experience even the “new” faces as familiar because of the same body odor was presented for all the faces both at the encoding and at the recognition task.

The analysis of odor ratings showed that the mask odor and the masked body odor were perceptually similar in intensity, arousal, valence and familiarity. This indicates that the masking procedure succeeded and that the effect of the masked body odor cannot be attributed to perceptual differences across odor conditions. This result supports previous evidence (Cecchetto, Lancini, Rumiati, & Parma, 2019; Parma et al., 2017) that human body odors modulate cognitive processes even when they are subliminally perceived, as it usually happens in our daily social life. Moreover, even though the two odor conditions were perceived as more intense, more arousing and more familiar than clean air, which confirmed that participants perceived an odor, they did not differ for pleasantness, which confirmed that the masker odor was neutral in pleasantness. This is an important aspect, since it suggests that the presented results did not emerge because of the valence of the odor.

### 4.2 Neural correlates of face recognition under human body odor cues

The analysis of brain activations for subsequent hits in the encoding task revealed that the interaction between odor conditions (odor vs. air) and groups (BO vs. MASK) were associated with an activation of the OFC and the dlPFC. This neuroimaging result may explain the obtained behavioral results. The dlPFC has been found to be involved in building relationships between items during successful memory encoding (Murray & Ranganath, 2007): dlPFC shows subsequent memory effects on tests that emphasize associative memory at encoding. OFC is considered the main secondary olfactory cortex (Seubert, Freiherr, Djordjevic, & Lundström, 2013) and receives the majority of the cortico‐cortical projections from the piriform cortex (the primary olfactory areas, Rolls, Critchley, & Treves, 1996). As such, OFC is thought to integrate the olfactory input with information coming from other sensory and cognitive processes to create a unitary experience (Gottfried & Dolan, 2003; Gottfried & Zald, 2005; Seubert, Freiherr, Djordjevic, & Lundström, 2013). Thus, activation within these two areas during the presentation of the masked body odor (but not during common odor or clean air) may indicate the combination of social information encoded in body odor with the visual information of faces during the encoding phase. This encoded body odor information is then used as a retrieval cue in the recognition phase.

This result adds a new perspective to previous findings (Reichert et al., 2017) by showing increased activation in the piriform cortex for successful encoding of stimuli when a congruent odor was presented during both encoding and recognition phases as compared to the incongruent presentation of odors. In the study by Reichert et al. (2017), there was no connection formed between the olfactory context and the visual stimuli. This might explain both the lack of increased memory performance for the congruent odor group compared to the incongruent odor group, but also the activation of the piriform cortex for successful encoding instead of the OFC as found in the present study. As discussed above, the piriform cortex, considered as the primary olfactory cortex, is particularly involved in odor detection tasks (Seubert, Freiherr, Djordjevic, & Lundström, 2013) and in learned associations between sensory cues (Li, Howard, Parrish, & Gottfried, 2008), while the OFC has been associated with higher‐order cognitive processes, including influences from other sensory modalities and cross‐modal associations (Gottfried & Dolan, 2003; Rolls & Baylis, 1994; Seubert, Freiherr, Djordjevic, & Lundström, 2013). In the previous study (Reichert et al., 2017), the missing association between the olfactory cortex and images might have prevented participants to create a unified memory representation.

In addition to the main interaction, we also investigated single contrasts (odor – air) in each group and across groups. In the encoding task, the subsequent hits were associated with activation in the putamen, piriform cortex, caudate nucleus, and the thalamus for the BO group (odor vs. air contrast). The putamen, caudate nucleus and the thalamus play an important role in learning and memory (Hélie, Ell, & Ashby, 2015) and in olfactory processing (Lundström et al., 2011). This result further supports our previous notion that already during the encoding phase visual stimuli were integrated with the olfactory information. Interestingly, significant activations in the piriform cortex, the putamen, and the hippocampus, another area involved in both olfactory processing (Gottfried & Dolan, 2003; Sobel et al., 2000) and memory (Spaniol et al., 2009), were found also for the contrast (odor vs. air) for the I‐BO group. However, no effects of body odor context were found on behavioral performance for the I‐BO group. A possible explanation for this result is that, even though the integration of faces with the body odor as contextual cue already happened in the encoding phase, the contextual cue is needed also in the recognition phase to effectively retrieve the unitary percept.

Moreover, the analysis of the encoding task for the contrast (odor – air) revealed that, across all groups, the hits were associated with activation in the pallidum, putamen, thalamus, and piriform cortex. Interestingly, the misses were associated only with pallidum and putamen, suggesting that the activation of the thalamus and the piriform cortex might be required for the successful integration of the olfactory information with faces and the subsequent successful recognition. While the role of the piriform cortex has already been proven during encoding‐recognition memory of cross‐modal information (Gottfried et al., 2004; Reichert et al., 2017), the role of the thalamus in olfactory processing is still unclear (Gottfried, 2010); yet some studies seem to suggest its involvement in associative learning (Tham, Stevenson, & Miller, 2009) and in olfactory attentional processing (Plailly, Howard, Gitelman, & Gottfried, 2008).

Finally, the analysis of the recognition phase in the contrast BO group vs. I‐BO group showed activation in occipital areas, and, in particular, activation in the fusiform face area, for both hits and false alarms. Since the odor and clean air conditions were included in these two analyses, these results can be attributed to the differences of congruency between the BO group and the I‐BO group. The increased activations in the fusiform face area and occipital areas for the congruent group might suggest an increased attention for face images when the odor context matched with the encoding phase. On the other side, in the congruent context, the presentation of the congruent information (body odor and face) only in the encoding or in the retrieval phase is not sufficient to facilitate the recognition of specific images, instead, it can activate general associations (when I see a face, I often smell a body odor too) for all the faces and not only to those presented at encoding.

### 4.3 Limitations

In the present study, for each participant of the BO and I‐BO groups, the same masked body odor (which consisted of the combination of four different donors) was used for all the faces in the odor block. This procedure could be one of the reasons why there were no significant results for the hit rates: the social information communicated through the body odor was not as specific as the visual information of each face image. Previous studies have shown that human body odors can communicate information regarding individuals' identity (e.g., age, gender, ethnicity, health status, sexual availability and personal predisposition; McClintock et al., 2005; Prokop‐Prigge, Greene, Varallo, Wysocki, & Preti, 2016; Wyatt, 2014) and emotional status (de Groot & Smeets, 2017; Wudarczyk et al., 2016). However, as combination of four different people, the information related to identity (e.g., age, health status) could have been misleading or confusing, while, since only emotionally neutral body odors where used, there was no specific information regarding the emotional status. Moreover, since the same odor was used for all the faces, there was not a peculiar body odor connected to a single identity. Since this was the first study to investigate the effects of body odor on the encoding and recognition of faces, we have preferred to apply an established paradigm (Cecchetto, Lancini, Rumiati, & Parma, 2019; Lundström, Boyle, Zatorre, & Jones‐Gotman, 2009; Mutic, Parma, Brünner, & Freiherr, 2015; Wudarczyk et al., 2016) with high consistency across trials. However, future studies should try to go beyond these limitations and clarify the effects of specific body odor presented in concomitant with the face donor. Furthermore, future studies need to investigate if the hereby shown effects are also present in men. This will also help to further clarify the nature of potential gender‐related effects of body odors in humans.

### 4.4 Conclusion

In conclusion, the present study provides evidence that specific odors can be particularly effective contextual cues for the encoding‐retrieval of related stimuli. In particular, we have shown that the social information presented through human body odors is integrated with the social information acquired during the encoding of faces and that this social olfactory cue presented in the recognition phase helps participants to recognize faces faster. This integration process is evident also at the neural level with the activation of OFC and dlPFC for the human body odor during successful encoding. Our results shed new light on the role of contextual odors during encoding and recognition of information and add new evidence that human body odors, even when unconsciously perceived because masked, are able to transmit social information that can be combined with other types of person‐identity information.

## Supporting information

Data S1. Materials and methods.

## Data Availability

The datasets generated and/or analyzed during the current study are available from the corresponding author on reasonable request.
