# Supplementary material for: Human body odor increases familiarity for faces during encoding‐retrieval task
Source: Hum Brain Mapp. 2020 Jan 6;41(7):1904–19. doi: 10.1002/hbm.24920 (PMC7268037; doi:10.1002/hbm.24920)
Supplement: Supplementary file 1 — Data S1. Materials and methods. [file HBM-41-1904-s001.docx]

**Supplementary material and methods**

**Participants’ menstrual cycle**

A cycle-length standardization formula was used to standardize the cycle length to a 28-day cycle by adjusting the follicular phase of each woman with respect to the length of her normal menstrual cycle. This process resulted in an invariant luteal phase, which remained constant at 14 days. If a woman was in her luteal phase, that is during the last 14 days of her cycle, her standardized cycle day was calculated by subtracting 28 days from the normal length of her cycle and adding the number counting from the first day of her cycle. Thus, if a woman had a 31-day cycle and it was the 23rd since the beginning of her cycle, her standardized cycle day would be calculated as follows: 28−31+23 = 20. If, instead, a woman was in her follicular phase, that is during the first 14 days of her cycle, her standardized cycle day was calculated by dividing her actual cycle day by the length of her normal cycle minus 14, multiplied by 14. If it was the 10th day after her period and her cycle was normally 34 days long, then the standardized-cycle-day would have be calculated as follows: [10/(34−14)]*14 = 7. For further details, refer to (Garver-Apgar et al., 2008).

**Odor pilot**

Before the main study, a pre-study was performed to determine which odor would be used as mask. Nineteen healthy female participants [Age, mean (SD) = 22.43 (2.15)] were asked to rate the intensity, familiarity, arousal and pleasantness of six olfactory stimuli. In three of these stimuli, a 250 µM concentration of estratetraenol (EcoChem, CAS: 1150-90-9) in 1,2-propylene glycol was mask with respectively 1) 1% cedarwood oil (Sigma-Aldrich, CAS: 68990-83-0) in 1,2-propylene glycol, 2) 1% vigoflor (International Flavors & Fragrances, New York, CAS 68480-11-5) in 1,2-propylene glycol, 3)1% eugenol (International Flavors & Fragrances, New York) in 1,2-propylene glycol. The other three stimuli consisted on the three solutions of 4) 1% cedarwood oil, 5) 1% vigoflor, 6) 1% eugenol without the estratetraenol. The six solutions were placed in identical brown bottles and coded. No significant differences were found between three odor solution with the estratetraenol and the respectively solution without the estratetraenol confirming the efficiency of the three odors as mask. However, eugenol was excluded because rated as significantly more familiar [mean(SD) = 6.94(1.84)] than cedarwood oil [mean(SD) = 4.36(1.86); *t*(36)=-4.29, *p*<0.001] and vigoflor [mean(SD) = 4.05(2.27); *t*(36)=-4.31, *p*<0.001] and significantly more pleasant [mean(SD) = 6.94(1.58)] than cedarwood oil [mean(SD) = 4.00(1.94); *t*(36)=-5.18, *p*<0.001] and vigoflor [mean(SD) = 4.15(1.97); *t*(36)=-4.80, *p*<0.001]. Finally, we decided to use vigoflor as mask because, even though not significantly different it was considered less familiar, more pleasant and less arousing [mean(SD) = 5.36(1.64)] than cedarwood oil [mean(SD) = 6.00(1.20)].

**Supplementary results**

**Table S1.** Mean values and standard deviations (in brackets) of intensity, familiarity, arousal and pleasantness ratings of groups for odor conditions and time points.

| Intensity rating | | | | |
| --- | --- | --- | --- | --- |
|  | Odor conditions | | | |
|  | Clean Air | | Odor | |
| Group | Pre | Post | Pre | Post |
| BO | 3.36 (1.86) | 3.63 (1.33) | 7.31 (1.89) | 6.21 (2.44) |
| MASK | 2.64 (1.55) | 3.17 (1.39) | 7.05 (1.67) | 7.35 (1.11) |
| I-BO | 3.44 (1.62) | 3.55 (1.13) | 7.72 (2.20) | 7.16 (1.38) |
| Familiarity rating | | | | |
|  | Odor conditions | | | |
|  | Clean Air | | Odor | |
| Group | Pre | Post | Pre | Post |
| BO | 4.21 (2.37) | 4.42 (1.95) | 5.79 (2.12) | 5.89 (2.15) |
| MASK | 2.94 (2.38) | 4.05 (2.38) | 5.17 (2.21) | 7.00 (1.66) |
| I-BO | 4.22 (2.36) | 4.44 (2.12) | 6.05 (2.36) | 6.44 (2.50) |
| Arousal rating | | | | |
|  | Odor conditions | | | |
|  | Clean Air | | Odor | |
| Group | Pre | Post | Pre | Post |
| BO | 4.16 (1.97) | 4.26 (1.93) | 6.58 (1.34) | 5.84 (1.74) |
| MASK | 3.64 (1.93) | 3.65 (1.49) | 5.94 (1.60) | 7.11 (1.26) |
| I-BO | 3.88 (1.68) | 3.94 (1.39) | 5.89 (1.90) | 6.05 (2.29) |
| Pleasantness rating | | | | |
|  | Odor conditions | | | |
|  | Clean Air | | Odor | |
| Group | Pre | Post | Pre | Post |
| BO | 6.05 (1.47) | 5.42 (2.05) | 5.26 (2.05) | 5.05 (2.12) |
| MASK | 6.35 (1.80) | 6.00 (2.26) | 4.58 (2.26) | 4.58 (2.37) |
| I-BO | 5.72 (1.64) | 5.78 (1.94) | 4.39 (1.94) | 4.94 (2.23) |

*Effects on mood*

No significant results were found from the LMM on STAI score with the interaction between groups and time points and with BDI score as fixed factors (all *β* < 2.41, all SE > 0.18, all t < 1.03, all p > .30). The LMM on positive PANAS score (AIC = 190.89; BIC = 214.86; logLik = -86.44; R2 = 0.70) showed a significant effect of group (*β* = -0.44, SE = 0.21, t = -2.07, p = 0.04): the group I-BO presents significant lower positive PANAS score than BO group. Moreover, a significant effect of time points (*β* = -0.38, SE = 0.13, t = -2.98, p = 0.004) was found with post task scores lower than pre task scores, and a significant effect of BDI (*β* = 0.04, SE = 0.02, t = 2.46, p = 0.017), higher scores of BDI present higher scores in positive PANAS. No significant results were found from the LMM on negative PANAS scores (all *β* < 0.01, all SE > 0.008, all t < 1.64, all p > 0.10). See Table S2 for means and standard deviations of PANAS and STAI scores.

**Table S2.** Mean values and standard deviations (in brackets) of PANAS and STAI scores.

| PANAS negative | | |
| --- | --- | --- |
| Group | Pre | Post |
| BO | 1.22 (0.18) | 1.15 (0.34) |
| MASK | 1.28 (0.22) | 1.21 (0.16) |
| I-BO | 1.26 (0.24) | 1.22 (0.51) |
| PANAS positive | | |
| Group | Pre | Post |
| BO | 3.15 (0.61) | 2.76 (0.73) |
| MASK | 3.13 (0.35) | 2.57 (0.54) |
| I-BO | 2.82 (0.85) | 2.46 (0.74) |
| STAI | | |
| Group | Pre | Post |
| BO | 33.42 (5.38) | 33.31 (5.67) |
| MASK | 36.88 (5.03) | 35.54 (8.05) |
| I-BO | 35.33 (6.38) | 36.44 (8.89) |
